# Indications and Outcomes of Patients Receiving Therapeutic Plasma Exchange under Critical Care Conditions: A Retrospective Eleven-Year Single-Center Study at a Tertiary Care Center

**DOI:** 10.3390/jcm12082876

**Published:** 2023-04-14

**Authors:** Alexander Ring, Wolfgang Alexander Sieber, Jan-Dirk Studt, Reto A. Schuepbach, Christoph Camille Ganter, Markus Gabriel Manz, Antonia Maria Susanne Müller, Sascha David

**Affiliations:** 1 Institute of Intensive Care Medicine, University Hospital Zurich, 8091 Zurich, Switzerland; alexander.ring@usz.ch (A.R.);; 2 Department of Medical Oncology and Hematology, University Hospital Zurich, 8091 Zurich, Switzerland

**Keywords:** therapeutic plasma exchange (TPE), intensive care unit (ICU), single-center study, ASFA classification

## Abstract

**Background:** Therapeutic plasma exchange (TPE) is frequently performed in critical care settings for heterogenous indications. However, specific intensive care unit (ICU) data regarding TPE indications, patient characteristics and technical details are sparse. **Methods**: We performed a retrospective, single-center study using data from January 2010 until August 2021 for patients treated with TPE in an ICU setting at the University Hospital Zurich. Data collected included patient characteristics and outcomes, ICU-specific parameters, as well as apheresis-specific technical parameters and complications. **Results**: We identified n = 105 patients receiving n = 408 TPEs for n = 24 indications during the study period. The most common was thrombotic microangiopathies (TMA) (38%), transplant-associated complications (16.3%) and vasculitis (14%). One-third of indications (35.2%) could not be classified according to ASFA. Anaphylaxis was the most common TPE-related complication (6.7%), while bleeding complications were rare (1%). The median duration of ICU stay was 8 ± 14 days. Ventilator support, renal replacement therapy or vasopressors were required in 59 (56.2%), 26 (24.8%), and 35 (33.3%) patients, respectively, and 6 (5.7%) patients required extracorporeal membrane oxygenation. The overall hospital survival rate was 88.6%. **Conclusion**: Our study provides valuable real-world data on heterogenous TPE indications for patients in the ICU setting, potentially supporting decision-making.

## 1. Introduction

Intensive care units (ICUs) are specialized treatment departments for patients requiring close monitoring and critical care, including pharmacological or mechanical organ support due to hemodynamic instability or organ failure. Despite improvements in diagnosis and treatment, mortality rates in ICUs remain high [1]. Therapeutic plasma exchange (TPE or PEX) is part of the intensivist armamentarium of life-saving therapeutic intervention for otherwise life-threatening conditions and is commonly used as a primary or adjunct therapy if clinically indicated [2,3,4].

TPE is an automated, extracorporeal process that allows the replacement of large volumes of plasma with a substitute solution (e.g., human albumin, healthy donor plasma, mixtures). The principal indications of TPE can be classified into two broad categories: (1) The removal of pathologic elements from circulation (e.g., autoantibodies, toxins, cytokines, adhesion molecules) via apheresis in order to reverse a pathologic process. Examples of this are (auto-) immune-mediated conditions, including neurological disorders, graft rejections following solid organ transplantation, or infectious diseases [3]. (2) The substitution of missing or inactivated protective plasma factors through healthy donor plasma. The classic example for this application is thrombotic thrombocytopenic purpura (TTP), a potentially fatal thrombotic microangiopathy (TMA), resulting from an acquired or inherited inefficacy of the von Willebrand factor cleaving protease ADAMTS13 (a disintegrin and metalloproteinase with a thrombospondin type 1 motif, member 13) and subsequent disseminated thrombotic events [5]. 

Separation of plasma and cellular components is achieved by centrifugal forces or membrane filtration [6]. While cellular components are usually returned to the patient, they can also be deliberately removed (e.g., in patients at risk of leukostasis) or harvested (i.e., cytapheresis) to perform hematopoietic stem cell transplantation [7,8]. 

The American Society for Apheresis (ASFA), supported by the European Society for Hemapheresis (ESFH), publishes regularly updated evidence-based treatment guidelines for TPE [3]. Only a limited number of studies have specifically addressed TPE in an ICU setting [9,10,11,12]. Here we address this limitation by performing a comprehensive, single-center 11-year retrospective analysis of TPE indications in critically ill patients at a tertiary care center.

## 2. Materials and Methods

### 2.1. Study Population and Research Design

We performed a retrospective, single-center study using data from January 2010 through September 2021 of consecutive patients treated with TPE at the Institute of Intensive Care Medicine (6 units, 72 beds total capacity) at the University Hospital Zurich (USZ). The key aim of our study was to analyze indications, technical details as well as patient- and ICU-specific parameters. Patients were identified from our electronic health record system (Klinikinformationssystem *KISIM*) using specific search terms: “plasmapheresis”, “plasma exchange” and/or “PEX”. We included all patients aged≥ 18 years treated for any indication of TPE in the ICU setting. Recorded and analyzed variables included patient socio-demographic data (age and gender), BMI, underlying disease (according to ASFA classification), major comorbidities, laboratory parameters, ICU-specific data (sequential organ failure assessment (SOFA) score, mechanical support (renal, ventilatory, circulatory), vasoactive-inotropic treatment, duration of ICU stay, hospital survival) and TPE-specific procedural parameters (number of TPE sessions, type and amount of replacement fluids, vascular access and complications).

Apheresis was performed in collaboration with the Department of Medical Oncology and Hematology using the COBE Spectra or Spectra Optia Apheresis System (Terumo, Tokyo, Japan), which applies continuous-flow centrifugation for the separation of cellular components from plasma. Plasma volumes were calculated using each patient’s weight and hematocrit value. Regional citrate anticoagulation was used in all cases.

The study was approved by the cantonal ethics committee Zurich (BASEC reference number 2021-01130) and performed in accordance with the Declaration of Helsinki.

### 2.2. Data Presentation

Raw data were processed using Excel (Microsoft, Redmond, WA, USA) or GraphPad Prism (GraphPad Software, San Diego, CA, USA). No power calculation was performed. Categorical variables are presented as counts and percentages, continuous variables are presented as mean and standard deviation (±SD) or percentage of the whole, as well as median and interquartile range. Two-sided unpaired students *t*-test or Mann–Withney test, and an alpha level below 5% was consider statistically significant.

## 3. Results

### 3.1. Study Population

We identified 105 patients that met the inclusion criteria for our study. The median number of patients treated per year throughout the study period was eight (range 3–18). The socio-demographic and other patient-specific data (gender, age, BMI, comorbidities) and ICU-specific variables (length of ICU stay, ventilation support, renal replacement therapy, extracorporeal membrane oxygenation/ECMO, vasoactive-inotropic treatment, sequential organ failure assessment/SOFA score) are presented in Table 1. The median age of our cohort was 56 years (range 17–85), with a similar distribution of female and male patients (50.5% vs. 49.5%, respectively). The most common comorbidities were hypertension (36.2%), obesity (defined as a BMI ≥ 30 kg/m^2^) (19%) and chronic kidney disease (CKD) (11.4%). 

### 3.2. TPE Indications and Laboratory Findings

**Figure 1:**
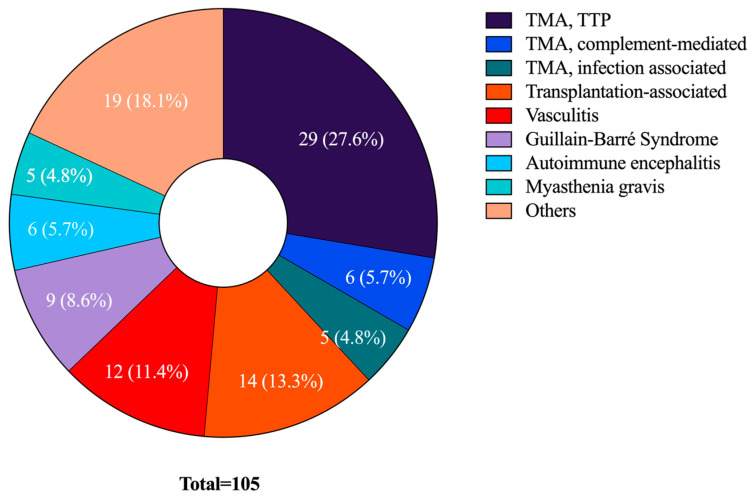
**Distribution of all indications for TPE in the ICU setting.** The indications (equal to afflicted patients) are shown in absolute numbers and percent of the total number of patients affected (n = 105). Abbreviations: TTP—Thrombotic thrombocytopenic purpura, TMA—Thrombotic microangiopathy. Others: acute liver failure (n = 2), macrophage activation syndrome (n = 1), progressive bulbar palsy (n = 1), idiopathic hypertriglyceridemia (n = 1), polytrauma-associated coagulopathy (n = 1), anti-IgLON5-associated encephalopathy (n = 1), systemic sclerosis (n = 1), chronic inflammatory demyelinating neuropathy (n = 1), postpartum microangiopathy (n = 1), Miller-Fisher Syndrome (n = 1), polyradiculoneuritis (n = 1), paraneoplastic neurological syndrome (n = 1), catastrophic antiphospholipid syndrome (n = 1), acute demyelinating neuropathy (n = 1), hypergammaglobulinemia (n = 1).

We identified 24 indications for TPE in the ICU setting in our institution (Table 2). Figure 1 illustrates the eight most common indications and their relative contribution to the whole cohort (n = 105) (Table 2). Expectedly, TMAs were the most documented conditions (n = 40, 46.5%), with TTP representing the most common TMA sub-entity (n = 29, 33.7%) (Table 2). Ten patients treated for TMA had normal ADAMTS13 activity or lack of inhibitor (one patient died during treatment and the analysis request was canceled).

The second most common indication for TPE transplantation-associated complications (n = 14, 16.3%). Of these, nine patients received TPE due to antibody-mediated rejection, three due to desensitization, one for rejection prophylaxis and one because of cellular rejection. Vasculitis (n = 12, 14%) ranked third in our analysis (11 ANCA-associated forms, 1 small vessel vasculitis without positive antibodies), with neurological indications including Guillain-Barré Syndrome (GBS) (n = 9, 10.5%), autoimmune encephalitis and myasthenia gravis representing other common indications.

In 100 (95.2%) patients, the indication for TPE could be assigned to the ASFA guidelines, with 56 (53.3%) representing category I (apheresis accepted as first-line treatment), 11 (10.5%) category III (individual decision making) and 33 (31.4%) with variable ASFA category depending on the subclassification (I or III) (Table 2, Supplementary Table S1) [3]. In order to display well-structured results, certain subclassifications with their own ASFA recommendation have been summarized and marked as “variable” ASFA classification and grade (transplantation-associated, vasculitis, all TMA). Five of the most common indications were classified as category I (TTP, GBS, autoimmune encephalitis, myasthenia gravis, Goodpasture’s syndrome), whereas one indication could only be assigned to category III (infection-associated TMA). Category III represents indications where evidence in the current body of literature is insufficient to clearly recommend TPE. Five (4.8%) patients could not be clearly classified based on ASFA recommendations (polytrauma-associated coagulopathy, anti-IgLON5-associated encephalopathy, polyradiculoneuritis, acute demyelinating neuropathy, autoimmune encephalitis) (Table 2).

Detailed analysis of clinical routine laboratory parameters showed anemia (mean hematocrit 0.27 ± 0.07 and hemoglobin levels 87 ± 25 g/L) in all and elevated inflammation parameters (n = 94) (CRP 76.7 ± 81.5 mg/L, procalcitonin 5.4 ± 18.3 µg/L, leukocyte count 13.2 ± 8.3 G/L) in most patients (n = 102, 97.1%) (Table 3). Leukocyte count was highest in patients with vasculitis (20.8 ± 12.9 G/L) and acute liver failure (n = 2) (22.8 ± 25.3 G/L), the latter also presenting with the most severe thrombocytopenia (31 ± 30 G/L) and the highest LDH levels (4070 ± 3789 U/L) (Table 3). Procalcitonin levels were highest in patients with transplantation-associated indications for TPE (25.1 ± 32.4 µg/L). More than half of the patients (n = 54) presented with kidney failure (estimated glomerular filtration rate (eGFR) 61 ± 40 mL/min, serum creatinine 210 ± 235 µmol/L) (Table 3), with the most severe impairment seen in patients with complement-mediated (12 ± 12 mL/min) and infection associated TMAs (n = 6) (15 ± 7 mL/min) (Table 3). Bilirubin levels were in elevated 38 patients (mean 28.1 ± 36 µmol/L) (Table 3). Statistical analysis of patients grouped by evidence for TPE according to ASFA guidelines showed no significant difference for all variables (Table 3) between patients and indication with clear ASFA category (I or III) and those that either variable or no ASFA category assigned (Supplementary Figure S1).

### 3.3. ICU-Specific Findings and Outcome

The median length of stay in the ICU across all patients and indications was 8 ± 14.4 days and the SOFA score was 7.7 ± 3.7 (on day 1 of TPE initiation) (Table 3). A total of 59 (56.2%) patients required ventilator support (52 (49.5%) invasive mechanical ventilation (IMV), three (2.9%) non-invasive ventilation (NIV) and four (3.8%) high-flow oxygen via nasal cannula). Additionally, 27 (25.7%) patients received renal replacement therapy and 35 (33.3%) vasoactive-inotropic support (Table 1 and Table 3). Six (5.7%) patients required extracorporeal membrane oxygenation (ECMO) (Table 1 and Table 3). The ICU survival rate was 88.6% in our cohort. Comparing ICU-specific parameters between patients with either ASFA category I or III indications of TPE with those presenting indications with either variable or unclear classification showed no statistically significant differences (Supplementary Figure S2). Numerically, patients with TTP and infection-associated TMAs showed a higher survival rate (95%) compared with complement-mediated TMAs (83.3%) (Table 4). All patients presenting Guillain-Barré Syndrome (n = 9) required invasive mechanical ventilation (IMV) and all survived their ICU stay (Table 4). The highest mean SOFA score on the first day of TPE recorded patients with liver failure (15 ± 4.2) and only patients (50%) survived the ICU (Table 4). Of note, 41 patients had no SOFA score available, which were recorded systematically only after 2013 in Switzerland. Patients with autoimmune encephalitis (n = 6) had the longest ICU stay (36 ± 29.9 days). Many of these patients required IMV (83.3%) and vasoactive-inotropic support (66.7%) and survival was below the mean (83.3%) (Table 4). Patients with Goodpasture’s syndrome (n = 3) had a poor outcome in our cohort (66.6% ICU mortality) (Table 4). 

### 3.4. TPE Procedural Details and Level of Evidence

**Figure 2:**
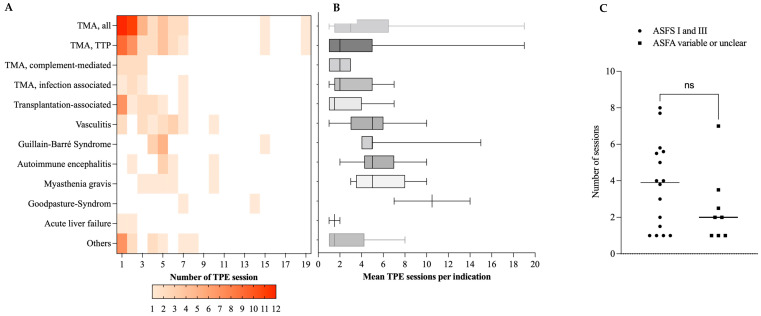
**Number of TPE sessions per indication.** (**A**) Heatmap showing the absolute number of TPE sessions per indication. Color coding represents mean session numbers (legend below graph). (**B**) Mean TPE sessions with SD. (**C**) Mean and SD of TPE session performed in patients with indications according to ASFA category. Abbreviations: TTP—Thrombotic thrombocytopenic purpura, TMA—Thrombotic microangiopathy, SD—standard deviation. Others: acute liver failure (n = 2), macrophage activation syndrome (n = 1), progressive bulbar palsy (n = 1), idiopathic hypertriglyceridemia (n = 1), polytrauma-associated coagulopathy (n = 1), anti-IgLON5-associated encephalopathy (n = 1), systemic sclerosis (n = 1), chronic inflammatory demyelinating neuropathy (n = 1), postpartum microangiopathy (n = 1), Miller-Fisher Syndrome (n = 1), polyradiculoneuritis (n = 1), paraneoplastic neurological syndrome (n = 1), catastrophic antiphospholipid syndrome (n = 1), acute demyelinating neuropathy (n = 1), hypergammaglobulinemia (n = 1). ns—not significant (*p* = 0.226) (Mann-Whitney test).

During the 11-year observation period, 408 TPE sessions (mean of 3.9 ± 3.3 per patient) were performed in the ICU setting (Table 2), representing 10.2% of all TPEs at the USZ during the study period (n = 4008). Figure 2A,B show the total number (A) and mean number (B) of TPE sessions for all indications. Patients presenting TTP were exchanged up to 19 times (Figure 2A) with a mean of 3.8 ± 4.2 session) (Figure 2B). TPE for TPA was discontinued after thrombocyte count reached >150 G/L.

**Figure 3:**
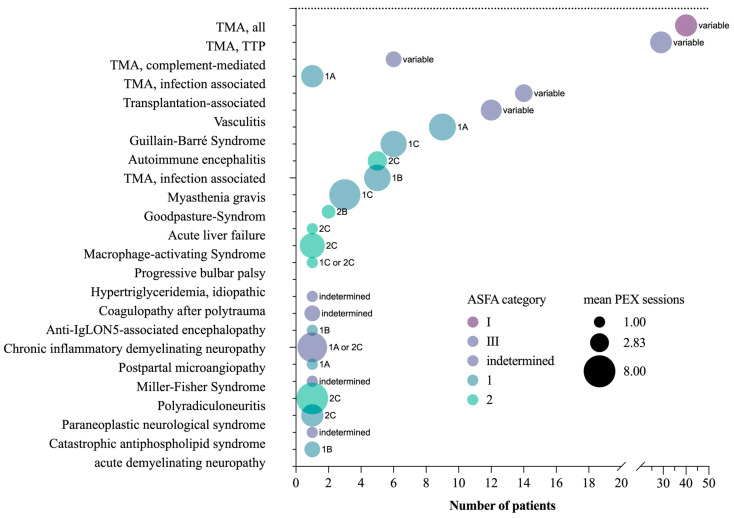
Combined analysis using indication for a number of patients, mean number of TPE sessions and ASFA category per TPE indication performed in an ICU setting. Circle color—ASFA category, circle size—mean number of TPE sessions per indication, text right to circles—ASFA grade (full data set available in Supplementary Table S1). Abbreviations: ASFA—American Society for Apheresis, TPE—therapeutic plasma exchange, TTP—Thrombotic thrombocytopenic purpura, TMA—Thrombotic microangiopathy, ICU—intensive care unit.

The highest mean number of TPE sessions was documented for Goodpasture’s syndrome (7 ± 5.3) (Figure 2A,B). Autoimmune-related diseases were exchanged every 2–3 days to allow transfer of IgG from tissues and subsequent removal from the bloodstream. The average number of sessions did not vary significantly between indications with clear ASFA recommendations (category I or III) and variable or no clear guideline-based indication (*p* = 0.226, Mann–Whitney test) (Figure 2C). Figure 3 summarizes the number of patients, mean TPE session count and ASFA category and grade of recommendation for each indication. 

The mean amount of replacement fluid was 3.4 ± 0.85 L per session, the type of replacement fluid was fresh frozen plasma (FFP) in 84 (80%) patients, human albumin in 13 (12.4%) patients and a mixture of both in five (4.8%) patients (Table 5). Three patient records (2.9%) did not specify the replacement fluid used. Most patients (n = 97, 92.4%) received a central vascular catheter for TPE and five (4.8%) had peripheral venous access (Table 5). Three (2.8%) had incomplete records.

Complications occurred in 10 (9.5%) patients and were mostly mild to moderate transfusion-related allergic reactions (grade 1 n = 4, 3.8%; grade 2 n = 3, 2.9%) that responded well to guideline confirm treatment (Table 5). Grade 1 represents reactions where symptoms either presented cutaneous, conjunctival, in the upper respiratory tract or others like nausea and metallic taste. The allergic reaction is classified as grade 2 when ≥2 of the organ systems listed in grade 1 are affected or if there are gastrointestinal symptoms present. More severe complications occurred in three patients (2.9%) (Table 5). One patient with progressive bulbar palsy suffered repeat bleeding while receiving human albumin and required substitution of fibrinogen, cryo-precipitated factor concentrates and Factor XIII. After switching the replacement fluid to FFP no further complications occurred. Another patient with TTP went into cardiac arrest and died during TPE. An autopsy revealed massive thrombi in both ventricles. The third patient developed atrial fibrillation, which was successfully treated with cardioversion. 

## 4. Discussion

Our retrospective study, spanning 11 years and including 105 patients treated with TPE in the ICU setting, demonstrated survival outcomes of 88.6% across all indications, reaffirming the role of TPE in the treatment of ICU patients. The spectrum of indications for TPE in the critically ill in our cohort was to a large degree comparable to common indications in non-ICU cohorts published previously, including TMA (TTP), catastrophic antiphospholipid syndrome, Guillain-Barré Syndrome or acute liver failure [2,3,4,13]. Other indications reported in the literature such as sepsis, PANDAS (pediatric autoimmune neuropsychiatric disorders associated with streptococcal infections) or drug overdoses/poisoning were not present in our cohort [14,15,16].

We found no statistically significant differences, including survival, between indications with clear ASFA grade and those with the variable or unclear grade, supporting the robustness of decision-making in centers with high TPE volume. Nevertheless, rigorous universal standards are still wanting. For example, TMAs represent a heterogeneous group of disorders characterized by similar clinical laboratory findings, but markedly different underlying pathophysiology [17]. The striking success of TPE as upfront emergency therapy in TTP, reducing morbidity and mortality from 90% in untreated patients to below 20%, led to broad application in other forms of TMA, independent of ADAMTS13 status [18,19]. In our study 11 (10.5%) patients received TPE for TMAs other than TTP, with normal ADAMTS13 activity or lack of inhibitor in nine (82%) cases. The underlying pathology for these entities may not be circulatory factors that can be removed by TPE, potentially limiting benefit or causing harm in these conditions [17,20]. A retrospective evaluation of TPE used during the EHEC-HUS pandemic in 2011 revealed a lack of efficiency of plasma exchange in enterotoxin-mediated HUS in adults [21]. On the other hand, current tests do not detect all ADAMTS13 deficiencies in patients presenting TTP and decision-making is additionally complicated by a lack of specific criteria [22]. Further studies are warranted, also regarding the management of refractory TMA cases (e.g., TPE regimens; combination with caplacizumab) [23,24], ideally in randomized trials. A growing number of indications of TPE further underline the potential and at the same time the necessity for standardization. Some examples include pediatric patients with H1N1 influenza-related respiratory failure and hemodynamic compromise, COVID-19 coagulopathy, septic shock or Ebola virus disease [25,26,27,28,29].

Previously reported TPE-associated complication rates vary significantly, ranging from 1–2% up to 39% [10,30,31]. Anaphylactic reactions were the primary complications in our cohort. Only a single bleeding event was found, which compares favorably with previous reports of up to 8.7% [10]. Hypotension, a common TPE complication correlating with low hematocrit, was not limiting in our study and was potentially masked by vasoactive-inotropic support [10].

In terms of procedural aspects, patients in our study received on average 3.9 ± 3.3 TPE sessions, like other studies in the ICU setting [10,11]. In contrast to other reports, several other indications (Guillain-Barré Syndrome (5.8 ± 3.5), autoimmune encephalitis (5.5 ± 2.6) and Goodpasture Syndrome (7.7 ± 6)) resulted in more TPE sessions than TMAs (3.5 ± 3.7) [10]. The choice of replacement fluid, which partially lacks solid standards, was predominantly FFP in our study, partially explained by the high percentage TTPs [30] and by the fact that albumin-based replacement strategies lead to a dilution coagulopathy bearing the additional risk of bleeding. One-third (n = 3) of GBS patients in our study also received FFP and one patient experienced atrial fibrillation during TPE with FFP, which was resolved with cardioversion. The use of FFP in patients with GBS has previously been associated with greater risks of citrate and transfusion reactions [30,32]. Still, the adverse advent rate for TPE in GBS was similar to the overall complication rate (was 11% vs. 9.5%). The median length of ICU stay (8 ± 14.4 days) compared favorably with previously reported outcomes [10,11,12]. Since cost and patient outcome are correlated with the length of ICU stay this difference is of importance and warrants further investigation of underlying factors [33].

The strength of our study lies in the detailed analysis of ICU disease indications and procedural aspects in a sizable number of critically ill patients (n = 105) receiving TPEs (n = 408). Limitations of our study include the single-center and retrospective character, limiting extrapolation and generalizability. Non-uniform documentation may introduce bias regarding, for example, minor complications and SOFA scores (which have only been recorded systematically in Switzerland after 2013). Additionally, the retrospective character of our analysis did not allow us to reliably distinguish a priori indications for ICU treatment against secondary factors leading to TPE in the ICU. Moreover, we have exclusively used a centrifugal plasma separation technique not allowing us to draw a generalized conclusion for membrane-based strategies.

## 5. Conclusions 

Our study provides comprehensive single-center data on TPE indications for patients treated in the ICU setting. Our finding of ICU survival of almost 90% of critically ill patients supports and strengthens the role of TPE in this setting. Favorable outcomes irrespective of unequivocal guideline support and the heterogeneous character of TPE applications in the ICU setting underscore the value of data from high-volume centers to support decision-making. Though limitations apply, we believe that our study might serve as a point of reference for similar institutions and might encourage similar research to enhance treatment standards and ultimately improve patient outcomes. 

## Figures and Tables

**Table 1:** **Patient characteristics.** Data are shown as patients per category and percentage of the total population. Abbreviations: COPD—chronic obstructive pulmonary disease, CAD—coronary artery disease, IMV—invasive mechanical ventilation, NIV—non-invasive ventilation, ECMO—extracorporeal membrane oxygenation. SOFA—Sequential Organ Failure Assessment. BMI—body mass index. ICU—intensive care unit. SD—standard deviation. TPE—therapeutic plasma exchange. * mean ± SD. ^#^ SOFA score was available for n = 64 (61%) and was only recorded after 2013.

Patient Characteristics n (%)	105 (100)
Gender	
*Female*	53 (50.5)
*Male*	52 (49.5)
Age	
*<18*	2 (1.9)
*18–29*	13 (12.4)
*30–49*	28 (26.7)
*50–64*	36 (34.3)
*65–79*	21 (20)
*80+*	5 (4.8)
*median age (range)*	56 (17–85)
BMI (kg/m^2^)	25.8 ± 4.8 *
Comorbidities	
*Obesity*	20 (19)
*Hypertension*	38 (36.2)
*Diabetes*	7 (6.7)
*COPD*	2 (1.9)
*Heart failure*	9 (8.6)
CAD	8 (7.6)
CKD	12 (11.4)
ICU specifics variables n (%)	
*Days of ICU stay*	14.5 ± 17.9
*non-ventilated*	46 (43.8)
*IMV*	52 (49.5)
*NIV*	3 (2.9)
*high flow*	4 (3.8)
*renal replacement*	27 (25.7)
*ECMO*	6 (5.7)
*Vasoactive-inotropic treatment*	35 (33.3)
*SOFA score ^#^, 1st day of TPE*	7.7 ± 3.7 *

**Table 2:** Data are shown as patients per category and percentage, as well as median TPE sessions with their respective interquartile range (IQR). Abbreviations: ASFA—American Society for Apheresis, TPE—therapeutic plasma exchange. I—Primary treatment, either stand-alone or in conjunction with other therapies, II—Secondary, treatment, either stand-alone or in conjunction with other therapies, III—Role of apheresis is uncertain, and decision-making should be individualized, IV—Evidence demonstrates apheresis to be ineffective or harmful.

Indication	Number of Patients n (%)	Median TPE Sessions (IQR)	ASFA Category	ASFA Grade
**All**	105 (100)	3 (4)	variable	variable
**TMA, all**	40 (38)	2 (3.25)	variable	variable
**TMA, TTP**	29 (27.6)	2 (4)	I	1A
**TMA, complement-mediated**	6 (5.7)	2 (1.5)	I or III	variable
**TMA, infection associated**	5 (4.9)	2 (1)	III	2C
**Transplantation-associated**	14 (13.3)	1.5 (2.75)	variable	variable
**Vasculitis**	12 (11.4)	5 (3)	variable	variable
**Guillain-Barré Syndrome**	9 (8.6)	5 (1)	I	1A
**Autoimmune encephalitis**	6 (5.7)	5 (0.75)	unclear	unclear
**Myasthenia gravis**	5 (4.9)	5 (2)	I	1B
**Goodpasture’s Syndrome**	3 (2.9)	7 (6)	I	1C
**Acute liver failure**	2 (1.9)	1.5 (0.5)	III	2B
**Macrophage-activation Syndrome**	1 (0.95)	1	III	2C
**Progressive bulbar palsy**	1 (0.95)	5	III	2C
**Hypertriglyceridemia, idiopathic**	1 (0.95)	1	III	1C or 2C
**Coagulopathy after polytrauma**	1 (0.95)	1	unclear	unclear
**Anti-IgLON5-associated encephalopathy**	1 (0.95)	2	unclear	unclear
**Systemic sclerosis**	1 (0.95)	4	I	1A
**Chronic inflammatory demyelinating neuropathy**	1 (0.95)	1	I	1B
**Postpartum microangiopathy**	1 (0.95)	7	I or III	1A or 2C
**Miller-Fisher Syndrome**	1 (0.95)	1	I	1A
**Polyradiculoneuritis**	1 (0.95)	1	unclear	unclear
**Paraneoplastic neurological syndrome**	1 (0.95)	8	III	2C
**Catastrophic antiphospholipid syndrome**	1 (0.95)	4	I	2C
**acute demyelinating neuropathy**	1 (0.95)	1	unclear	unclear
**Hyperviscosity in hypergammaglobulinemia**	1 (0.95)	2	I	1B

**Table 3:** **Laboratory parameters before initiation of TPE per indication**. Data are presented as median and interquartile range. Abbreviations: TTP—Thrombotic thrombocytopenic purpura, TMA—Thrombotic microangiopathy, TPE—therapeutic plasma exchange, eGFR—estimated glomerular filtration rate. ^#^ Transplantation-associated included: n = 3 desensitization, n = 9 antibody- mediated rejections, n = 1 cellular rejection, n = 1 rejection prophylaxis.

Indication (n)	Hematocrit	Hemoglobin (g/L)	Leukocytes (G/L)	Thrombocytes (G/L)	CRP (mg/L)	Procalcitonin (µg/L)	LDH (U/L)	Bilirubin (µmol/L)	Creatinine (µmol/L)	eGFR (mL/min)
All (105)	0.25 (0.08)	83 (24)	11.1 (10.5)	138 (225)	48 (84)	0.6 (1.25)	764 (1293)	15 (27.8)	116 (199)	57 (72)
TMA, all (40)	0.23 (0.09)	78 (28)	10.4 (7.7)	37 (69)	57 (93)	0.81 (1.25)	1650 (1713)	19 (35)	200 (221)	23 (60)
TMA, TTP (29)	0.23 (0.09)	78 (31)	9.9 (5.9)	31 (92)	45.5 (86.3)	0.51 (1.4)	1274 (1716)	31.5 (47.5)	156 (187)	34 (70)
TMA, complement-mediated (6)	0.21 (0.04)	73 (10)	14.2 (3.1)	43 (39)	50 (29)	1.17 (0.46)	1779 (400)	19 (3)	619 (401)	7 (3)
TMA, infection associated (5)	0.28 (0.09)	96 (26)	15.2 (5.6)	27 (41)	100 (48)	1.31 (0.43)	2697 (1297)	15 (2)	282 (43)	16 (5)
Transplantation-associated ^#^ (14)	0.28 (0.12)	89 (43)	12.4 (12.4)	140 (83)	26.5 (47.8)	1 (5.31)	794 (462)	9 (26)	184 (202)	29 (70)
Vasculitis (12)	0.27 (0.04)	85 (14)	18.1 (7.2)	379 (188)	114.5 (125.5)	1.19 (2.17)	543 (93)	8 (5)	188 (282)	30 (63)
Guillain-Barré Syndrome (9)	0.29 (0.12)	96 (36)	8.9 (4.7)	270 (95)	37 (25)	0.22 (0.08)	372 (79)	6.5 (7)	60 (28)	93 (15)
Autoimmune encephalitis (6)	0.27 (0.04)	85 (17)	8.6 (6.7)	201 (133)	18 (14.3)	0.1 (0.05)	343 (157)	3 (0.8)	59 (57)	111 (56)
Myasthenia gravis (5)	0.35 (0.16)	118 (61)	10.3 (5.2)	305 (37)	16 (19.5)	0.98 (1.31)	337 (9)	9 (3)	67 (31)	105 (39)
Goodpasture’s Syndrome (3)	0.23 (0.09)	76 (29)	17.4 (5.9)	285 (27.5)	47 (14)	3.2	381(108)	21 (4)	190 (55)	42 (32)
Acute liver failure (2)	0.29 (0.08)	92 (25)	22.8 (17.9)	31 (22)	81.5 (49.5)	0.36 (0.12)	4070 (2686)	78.5 (34.5)	90 (7)	86 (11)
Macrophage-activation syndrome (1)	0.254	85	15.81	55	133	4.43	3297	57	167	39
Progressive bulbar palsy (1)	0.223	68	5.92	249	39	0.37	204	4	52	84
Hypertriglyceridemia, idiopathic (1)	0.191	64	28.83	154	113	8.91	698	122	99	68
Polytrauma-associated coagulopathy (1)	0.135	45	1.78	89	0.6	0.1	383	6	73	97
Anti-IgLON5-associated encephalopathy (1)	0.356	107	11.13	178	91	0.09	-	-	71	90
Systemic sclerosis (1)	0.261	85	20.06	135	47	0.43	1674	36	266	16
Chronic inflammatory demyelinating neuropathy (1)	0.3	96	5.67	214	1.8	0.5	221	-	58	111
Postpartum microangiopathy (1)	0.21	77	17.96	55	81	-	1692	10	283	18
Miller-Fisher Syndrome (1)	0.257	87	11.18	225	131	0.48	-	5	51	107
Polyradiculoneuritis (1)	0.235	78	23.55	444	16	0.17	543	-	75	90
Paraneoplastic neurological syndrome (1)	0.316	115	10.92	158	69	0.5	395	13	50	90
Catastrophic antiphospholipid syndrome (1)	0.271	93	20.99	99	164	0.96	788	4	103	61
acute demyelinating neuropathy (1)	0.249	86	18.59	299	98	0.07	-	11	39	90
Hypergammaglobulinemia (1)	0.26	88	11.9	36	38	0.67	715	100	147	44

**Table 4:** **Indications with ICU specifics.** Data are shown as patients per category and percentage of the total population. Abbreviations: TTP—Thrombotic thrombocytopenic purpura, TMA—Thrombotic microangiopathy, SOFA score—Sequential Organ Failure Assessment score, ICU—intensive care unit, ECMO—extracorporeal membrane oxygenation, TPE—plasma exchange, SD—standard deviation. * Data expressed as median and interquartile range ^#^ Transplantation-associated included: n = 3 desensitization, n = 9 antibody-mediated rejections, n = 1 cellular rejection, n = 1 rejection prophylaxis.

Indications (n, % within Indication)	Non-Ventilated	IMV	NIV	High Flow	Renal Replacement	ECMO	Vaso-pressors	Length of ICU Stay *	Length of Hospital Stay *	ICU Survival	SOFA Score ICU Submission *	SOFA Score 1st Day TPE *
All (105)	46 (43.8)	52 (49.5)	3 (2.9)	4 (3.8)	27 (25.7)	6 (5.7)	35 (33.3)	8 (14)	29 (35)	93 (88.6)	7 (3.3)	7 (5)
TMA, all (40)	32 (80)	7 (17.5)	0	1 (2.5)	10 (25)	1 (2.5)	5 (12.5)	4 (5.3)	13 (18.3)	36 (90)	8 (2.5)	8 (2.5)
TMA, TTP (29)	24 (82.8)	5 (17.2)	0	0	5 (17.2)	1 (3.5)	2 (6.9)	4 (6)	13 (22)	26 (89.7)	7.5 (3)	7.5 (3)
TMA, complement-mediated (6)	4 (66.7)	2 (3.3)	0	0	2 (3.3)	0	1 (16.7)	4 (3.8)	13.5 (11.5)	5 (83.3)	8 (1.5)	8 (1.5)
TMA, infection associated (5)	4 (80)	0	0	1 (20)	3 (60)	0	2 (40)	5 (3)	13 (23)	5 (100)	10 (3)	10 (3)
Transplantation-associated ^#^ (14)	6 (42.9)	7 (50)	1 (7.1)	0	8 (57.1)	4 (28.6)	7 (50)	15(25)	51 (34.8)	12 (85.7)	7 (3)	7 (4)
Vasculitis (12)	2 (16.7)	7 (58.3)	2 (16.7)	1 (8.3)	3 (25)	1 (8.3)	6 (50)	7 (6)	25.5 (15.3)	12 (100)	6 (2)	6 (2)
Guillain-Barré Syndrome (9)	0	9 (100)	0	0	0	0	2 (22.2)	15 (20)	29 (8)	9 (100)	5 (4)	4 (2)
Autoimmune encephalitis (6)	1 (16.7)	5 (83.3)	0	0	0	0	4 (66.7)	31.5 (28.8)	79.5 (36.5)	5 (83.3)	6 (3)	7.5 (3.8)
Myasthenia gravis (5)	0	5 (100)	0	0	0	0	3 (60)	20 (5)	48 (25)	5 (100)	5.5 (4.5)	4 (4)
Goodpasture’s Syndrome (3)	0	2 (66.7)	0	1 (33.3)	0	0	2 (66.7)	18 (24)	29 (26.5)	2 (66.7)	9 (2.5)	9 (2.5)
Acute liver failure (2)	0	1 (50)	0	1 (50)	2 (100)	0	1 (50)	7.5 (1.5)	15.5 (9.5)	1 (50)	11.5 (0.5)	15.(3)
Macrophage-activating Syndrome (1)	0	1 (100)	0	0	1	0	1 (100)	4	25	0	18	16
Progressive bulbar palsy (1)	0	1 (100)	0	0	0	0	0	17	55	1 (100)	9	4
Hypertriglyceridemia, idiopathic (1)	0	1 (100)	0	0	1	0	1 (100)	29	61	1 (100)	12	13
Coagulopathy after polytrauma (1)	0	1 (100)	0	0	-	-	1 (100)	2	2	0	12	12
Anti-IgLON5-associated encephalopathy (1)	0	1 (100)	0	0	0	0	0	9	35	1 (100)	7	3
Systemic sclerosis (1)	1 (100)	0	0	0	1 (100)	0	0	11	35	0	8	8
Chronic inflammatory demyelinating neuropathy (1)	0	1 (100)	0	0	0	0	1 (100)	31	75	1 (100)	6	4
Postpartum microangiopathy (1)	1 (100)	0	0	0	0	0	0	12	45	1 (100)	-	-
Miller-Fisher Syndrome (1)	0	1 (100)	0	0	0	0	0	28	55	1 (100)	-	-
Polyradiculoneuritis (1)	0	1 (100)	0	0	0	0	0	30	35	1 (100)	-	-
Paraneoplastic neurological syndrome (1)	0	1 (100)	0	0	0	0	1 (100)	17	34	1 (100)	-	-
Catastrophic antiphospholipid syndrome (1)	1 (100)	0	0	0	0	0	0	6	25	1 (100)	-	-
acute demyelinating neuropathy (1)	1 (100)	0	0	0	0	0	0	6	90	1 (100)	-	-
Hyperviscosity in hypergammaglobulinemia (1)	1 (100)	0	0	0	1 (100)	0	0	5	51	1 (100)	-	-

**Table 5:** **TPE-specific parameters**. Data are shown as patients per category and percentage of the total population. * Data expressed as mean ± SD. Abbreviations: FFP—fresh frozen plasma, TPE—therapeutic plasma exchange. SD—standard deviation.

**TPE sessions**	**n**
*total TPE sessions*	408
*average TPE sessions per patient*	3.9 ± 3.3 *
**Replacement fluid**	**n (%)**
*amount per session (L)*	3.4 ± 0.85 *
*FFP*	84 (80)
*Human albumin*	13 (12.4)
*Mix*	5 (4.8)
**Vascular access**	**n (%)**
*central*	97 (92.4)
*periphery*	5 (4.8)
**Complications**	**n (%)**
*transfusion reaction, grade 1 allergic reaction*	4 (3.8)
*transfusion reaction, grade 2 allergic reaction*	3 (2.9)
*bleeding complications*	1 (0.95)
*others*	2 (1.9)

## Data Availability

All data generated or analyzed during this study are included in this published article [and its Supplementary Information Files].

## Supplementary Materials

The following supporting information can be downloaded at: https://www.mdpi.com/article/10.3390/jcm12082876/s1, Figure S1: Statistical analysis of patients grouped by evidence for TPE according to ASFA guidelines with clear ASFA category (I or III) compared with either variable or no ASFA category assigned. Unpaired student’s *t*-test. ns—not significant (Mann-Whitney test); Figure S2: Statistical analysis comparing ICU-specific parameters between patients with either ASFA category I or III indications for TPE with those presenting with indications with either variable or unclear classification. Unpaired student’s *t*-test. ns—not significant (Mann–Whitney test); Table S1: ASFA Category Definitions for Therapeutic Plasma Exchange, Journal of Apheresis.

## Abbreviations

TPE	therapeutic plasma exchange
ICU	intensive care unit
TTP	Thrombotic thrombocytopenic purpura
TMA	Thrombotic microangiopathy
ASFA	American Society for Apheresis
ESFH	European Society for Hemapheresis
ECMO	extracorporeal membrane oxygenation
SD	standard deviation
COPD	chronic obstructive pulmonary disease
CAD	coronary artery disease
IMV	invasive mechanical ventilation
NIV	non-invasive ventilation
SOFA score	Sequential Organ Failure Assessment score
FFP	fresh frozen plasma
