# Supplementary material for: Indications and Outcomes of Patients Receiving Therapeutic Plasma Exchange under Critical Care Conditions: A Retrospective Eleven-Year Single-Center Study at a Tertiary Care Center
Source: J Clin Med. 2023 Apr 14;12(8):2876. doi: 10.3390/jcm12082876 (PMC10141205; doi:10.3390/jcm12082876)
Supplement: Supplementary file 1 [file jcm-12-02876-s001.zip › jcm-2322804-supplementary.pdf]

Journal of
Clinical Medicine

MDPI

Supplementary materials

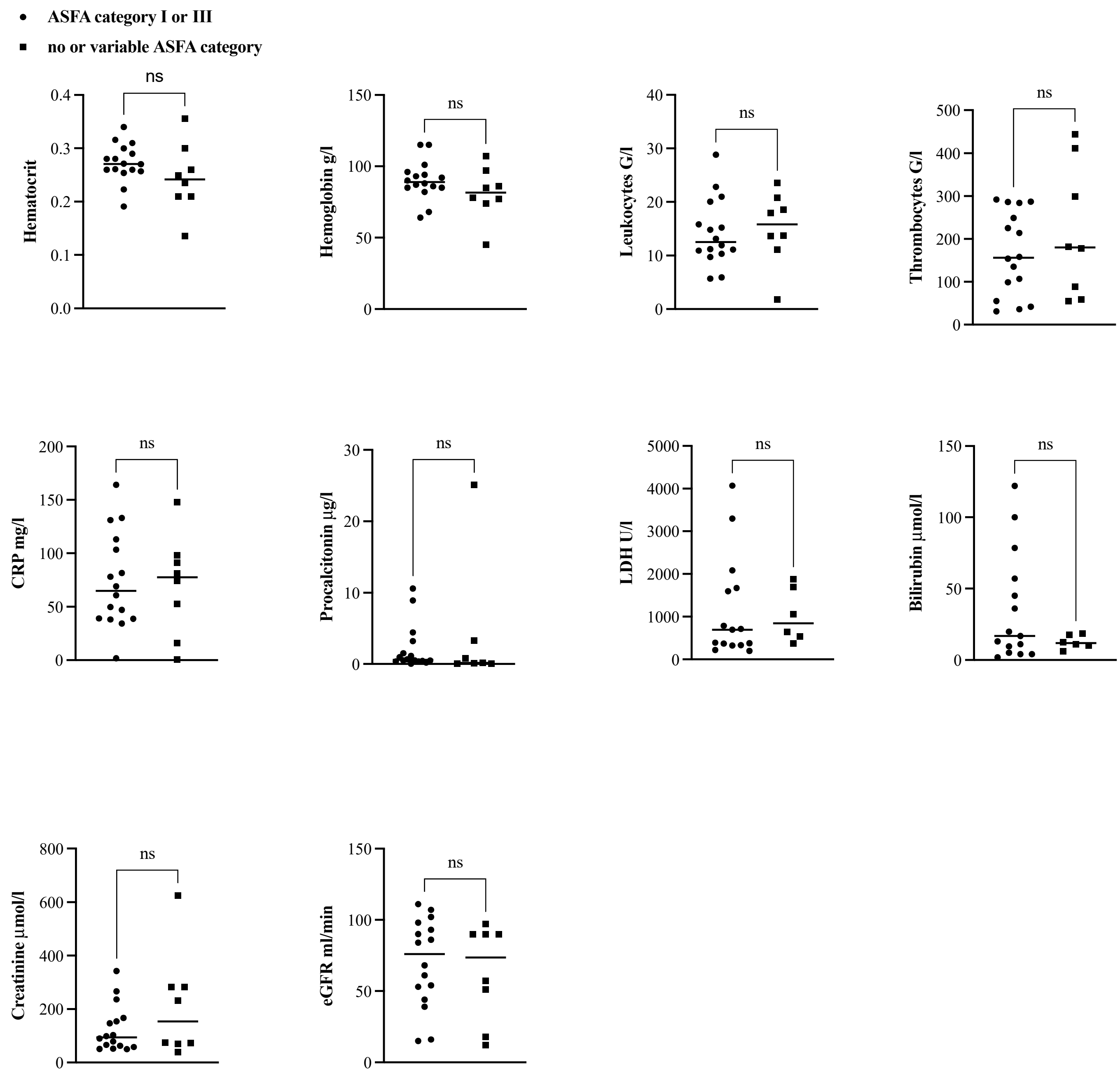

Supplementary figure S1: Statistical analysis of patients grouped by evidence for TPE according to ASFA guidelines with clear ASFA category (I or III) compared with either variable or no ASFA category assigned. Unpaired student's t test. ns – not significant (Mann-Whitney test).

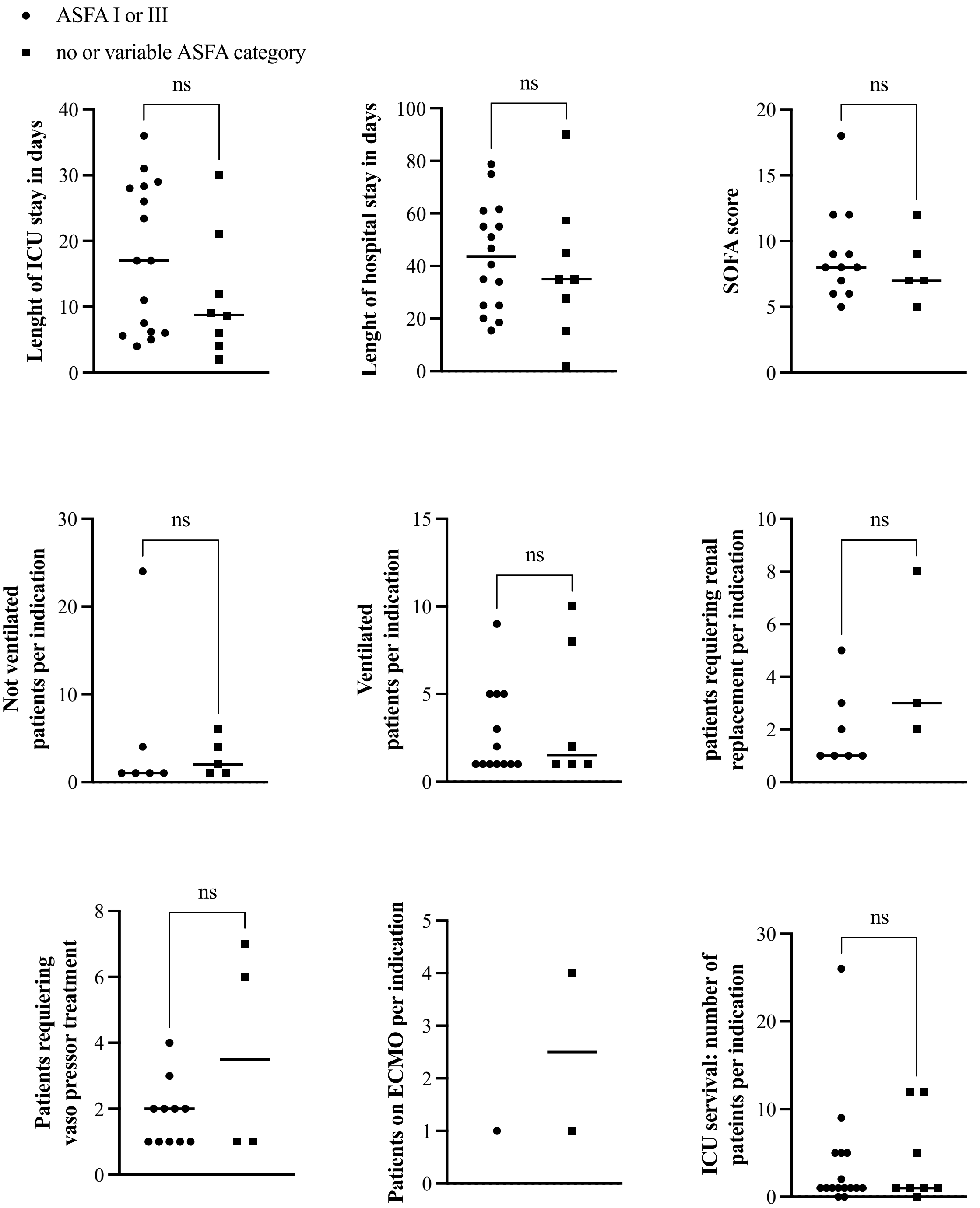

Supplementary figure S2: Statistical analysis comparing ICU specific parameters between patients with either ASFA category I or III indications for TPE with those presenting with indications with either variable or unclear classification. Unpaired student's t test. ns – not significant (Mann-Whitney test).

Supplementary table S1: ASFA Category Definitions for Therapeutic Plasma Exchange, Journal of Apheresis [3]

Category	Description
I	Disorders for which apheresis is accepted as first-line therapy, either as a primary standalone treatment or in conjunction with other modes of treatment.
II	Disorders for which apheresis is accepted as second-line therapy, either as a standalone treatment or in conjunction with other modes of treatment.
III	Optimum role of apheresis therapy is not established. Decision making should be individualized.
IV	Disorders in which published evidence demonstrates or suggests apheresis to be ineffective or harmful. IRB approval is desirable if apheresis treatment is undertaken in these circumstances
